# Raman Spectroscopy and Cystic Fibrosis Disease: An Alternative Potential Tool for Cystic Fibrosis Transmembrane Conductance Regulator (CFTR) Modulator Response Differentiation—A Pilot Study Based on Serum Samples

**DOI:** 10.3390/molecules29020433

**Published:** 2024-01-16

**Authors:** Giuseppe Acri, Barbara Testagrossa, Maria Cristina Lucanto, Simona Cristadoro, Salvatore Pellegrino, Elisa Ruello, Stefano Costa

**Affiliations:** 1 Dipartimento di Scienze Biomediche, Odontoiatriche, e delle Immagini Morfologiche e Funzionali, Università degli Studi di Messina, 98125 Messina, Italy; giuseppe.acri@unime.it (G.A.); eruello@unime.it (E.R.); 2 Unità Operativa Semplice Dipartimentale Gastroenterologia Pediatrica e Fibrosi Cistica, Azienda, Ospedaliera Universitaria Policlinico G. Martino, Via Consolare Valeria, 98125 Messina, Italy; mariacristina.lucanto@polime.it (M.C.L.); simona.cristadoro@polime.it (S.C.); salvatore.pellegrino@polime.it (S.P.); stefano.costa@polime.it (S.C.)

**Keywords:** Raman spectroscopy, cystic fibrosis, lipids biomarkers, Voigt deconvolution

## Abstract

Cystic fibrosis (CF) is a genetic disorder that alters chloride transport in mucous membranes. Recent studies have demonstrated that treatment with modulators of the chloride channel reduces inflammatory markers, restoring, among others, the imbalance of lipids. In this study, we analyzed the serum samples of treated and non-treated patients with modulators with Raman spectroscopy. Nineteen (eight treated an eleven non-treated) patients were considered. The main difference between the two groups appeared in the 3020–2800 cm^−1^ range. A Voigt deconvolution fit was performed, and nine sub-bands were identified. To distinguish between treated and non-treated patients, the area ratio between the CH_3_ and CH_2_ vibration modes was calculated for each patient. The results were validated using statistical analyses. In particular, receiver operating characteristic (ROC) curves and Youden index (Y) were calculated (Area Under Curve (AUC): 0.977; Y: 3.30). An ROC curve represents the performance of the classification, illustrating the diagnostic ability of Raman spectroscopy. It was demonstrated that Raman spectroscopy is able to highlight peculiar differences between elexacaftor/tezacaftor/ivacaftor (ETI)-treated and non-treated patients, in relation with lipids biomarkers.

## 1. Introduction

Cystic fibrosis (CF) is a genetic disorder caused by cystic fibrosis transmembrane conductance regulator (CFTR) gene mutations. This results in defective or insufficient CFTR protein and altered chloride transport in mucous membranes throughout the body, consequently causing salt and water dysregulation, and electrolyte imbalance, and progressive damage within multiple organs systems such as the respiratory system, the pancreas, the gastrointestinal tract, and the reproductive system [1,2]. CF is present in all ethnicities with an incidence varying from ~1:1000 to ~1:30,000 live births depending on the genetic ancestry of the population.

The CFTR protein is a membrane ATP-binding cassette (ABC) transporter-class ion channel that conducts chloride and bicarbonate ions across epithelial cell membranes, and it is regulated by cyclic adenosine monophosphate (cAMP) and protein kinase A phosphorylation [3,4]. Different mutations lead to a variety of malfunctions in the CFTR protein, ranging from folding defects (leading to decreases in anion transport) to complete absence of mature proteins due to premature degradation of aberrant proteins or stop codon mutations (nonsense mutations) that results in a shorter, unfinished protein product. The CFTR protein is predominantly expressed in epithelial cells and serves as a fundamental ion and water regulator in different organs [2]. Generally, the severity of the protein disfunction correlates with the severity of clinical disease [5].

CFTR modulators are a heterogeneous group of drugs that bind the CFTR protein in specific sites either during or after protein processing. There are several categories of therapies that may interact with the CFTR protein to increase its function. These include potentiators, correctors, read-through agents, amplifiers, and stabilizers [6]. Potentiators and correctors are clinically available, while the development of the other categories of molecules is still under study. Potentiators enhance or restore ion channel activity and thereby improve chloride and bicarbonate transport across the CFTR protein. This class of drug is particularly effective for those mutations affecting the stability or the opening of the channel in the cell membrane, where the primary protein defect is related to ion channel dysregulation. Correctors bind to the immature CFTR protein and improve protein folding, processing, and trafficking to the cellular membrane. This class of drug has been extensively tested with specific mutations, such as F508del (the most frequent CFTR mutation). Of these, different combinations of potentiators (e.g., ivacaftor) and correctors (e.g., lumacaftor, tezacaftor, elexacaftor) have now been approved and are commercially available; this has revolutionized the lives of people with CF, changing the disease trajectory and improving the clinical outcomes [7,8,9,10].

In particular, the triple combination of CFTR modulators (elexacaftor/tezacaftor/ivacaftor, or ETI) was approved by the Food and Drug Administration through fast-track and orphan drug study status on October 21, 2019, for people with at least one copy of the F508del mutation [10]. After approval, therapy became quickly available all over the world for the majority of people with CF, initially for patients aged 12 or over and shortly after for patients in the 6–11 age group. ETI was reported as safe and highly effective in two large randomized clinical trials, with clear evidence of improving multiple clinical outcomes at 4, 24, and 48 weeks [11,12,13,14,15,16,17]. ETI is administered orally. The recommended dose is two tablets (each containing elexacaftor 100 mg/tezacaftor 50 mg/ivacaftor 75 mg) taken in the morning and one tablet (containing ivacaftor 150 mg) taken in the evening, approximately 12 h apart. It is suggested to take ETI together with a fat-containing meal in order to improve drug absorption [10].

Beyond the clinical results obtained by ETI treatment, its impact on the inflammatory status remains unclear. in vitro demonstrations have shown that ETI combination partially restores lipid imbalance (i.e., abnormalities in fatty acid, ceramides and cholesterol metabolism) [18]. Veltman et al. reported that elevated oxidative stress, an abnormal lipid fingerprint, and enhanced pro-inflammatory signaling in well-differentiated bronchial epithelial cells isolated from neonatal CFTR KO pigs and adult CF patients undergoing lung transplantation were partially corrected after incubation with a combination of ETI and antioxidants [18]. More recently, a study conducted by De Vuyst et al. showed that ETI treatment reduced inflammatory markers and positive bacterial cultures on bronchoalveolar lavage in patients with CF [19]. In order to verify if the differences in the molecular responses between treated and non-treated patients were due to ETI treatment, Raman spectroscopy was used as a non-invasive methodology and serum samples were analyzed.

Raman spectroscopy, a vibrational technique, is a powerful analytic modality widely used in the study of complex biological samples [20]. In Raman spectroscopy, a sample is irradiated by a laser beam and the resultant scattered light is observed [21,22]. The Raman spectra are very sensitive to the structure, conformation and environment of the molecules [23]. This methodology has been proposed as a valid alternative tool in clinical diagnosis [24]. It has been applied to detect different diseases [24,25] as well as protein and lipid identification to investigate metabolic changes [26,27,28,29].

In recent years, there have been numerous studies related to biofluid analysis using vibrational spectroscopy [23,30,31,32]; this is because it requires minimal sample preparation and is easy to apply.

This study, based on Raman spectroscopy, was conducted on blood serum samples of ETI-treated and non-treated patients to differentiate the ETI response, thus confirming the treatment efficacy.

The reliability of the proposed experimental methodology was evaluated by conducting statistical analyses. In particular, the receiver operating characteristic (ROC) curve and Youden index (Y) were used, with its associated cut-off point was determined.

## 2. Results

**Figure 1:**
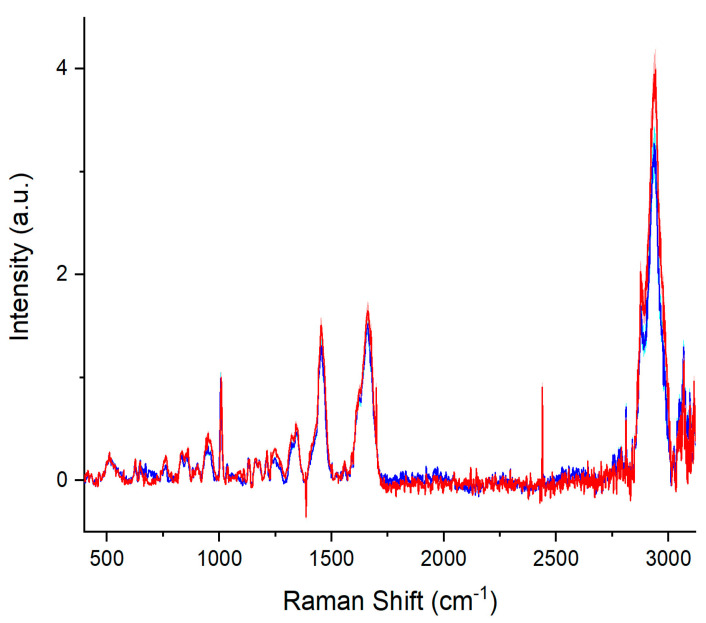
Average Raman spectra and SD of the sera of ETI-treated patients (blue line—SD cyan area) and non-treated patients (red line—SD light red area).

In Figure 1, the average Raman spectra and standard deviation (SD) of serum samples of the ETI-treated (blue line, SD: cyan area) and non-treated (red line, SD: light red area) patients are shown after baseline corrections and normalization to the phenylalanine peak. The spectra show the main features assigned to the serum constituents [33,34,35,36,37,38,39,40,41,42].

**Figure 2:**
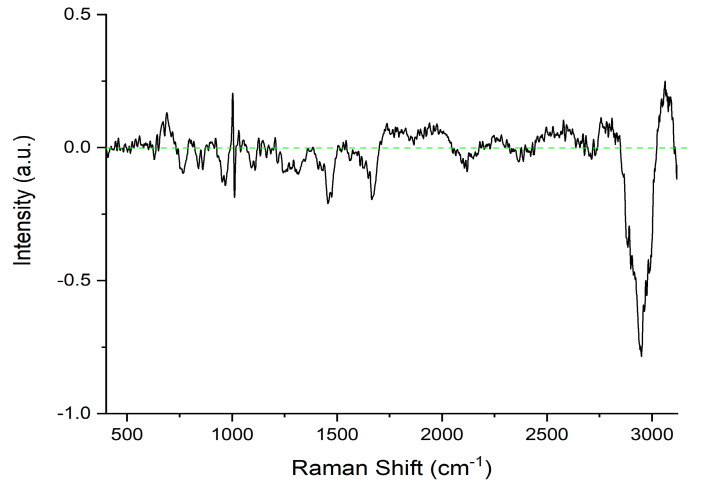
The difference spectrum of the CF patients. The difference spectrum was computed by subtracting the average spectrum of the ETI-treated patients from the average spectrum of the non-treated patients. The green line represents the zero line.

To better compare the two different sets of Raman data, a spectral subtraction between the average Raman spectra of the serum samples of ETI-treated and non-treated patients was performed. The difference spectrum is reported in Figure 2. The main differences between the ETI-treated and non-treated average spectra appear in the 3020–2800 cm^−1^ spectral range (center of the Raman band: about 2935 cm^−1^). As reported in the literature, this band can be attributed to C-H anti-symmetric and symmetric stretching vibration modes [41,43]. The above spectral modifications, induced by an inflammatory state, could be associated with changes in the secondary structure.

**Figure 3:**
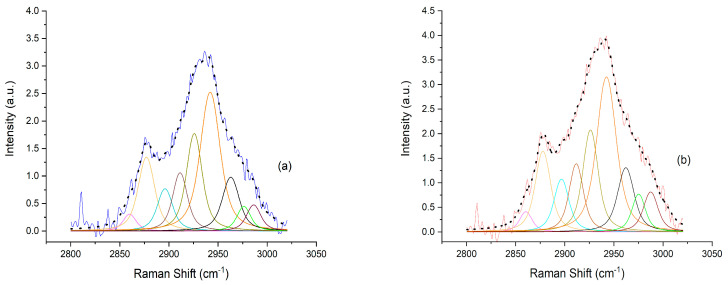
The Voigt deconvolution of the 3020–2800 cm^−1^ band. Nine sub-bands are present in both the ETI-treated patients (**a**) and non-treated patients (**b**). The straight line represents the original spectrum (blue for ETI-treated patients (**a**); red for non-treated patients (**b**)), whereas the black dotted lines in both spectra show the composite spectrum obtained from the deconvolution computation. The identified nine sub-bands are shown in the following colors: purple, yellow, cyan, brown, olive green, orange, black, green and reddish purple.

For this reason, the behavior of the 3020–2800 cm^−1^ band was analyzed using a Voigt fit. Nine components were identified theoretically in a first step by using the second derivative analysis and were experimentally identified by the Voigt deconvolutions. The deconvoluted spectra of the serum samples of the ETI-treated and non-treated patients are reported in Figure 3a and Figure 3b, respectively. In these Figures, all the sub-bands that contribute to the Raman spectrum are included, as identified using the Omnic software (OMNIC for Dispersive Raman 9.1.24).

In Table 1, we indicate the Raman peak positions of all the sub-bands and their tentative assignment based on the literature.

From the tentative assignment, as reported in Table 1, the sub-bands can be associated with the CH_3_ and CH_2_ symmetric and anti-symmetric stretching of lipids. This is due to the fact that lipids in serum are composed by different compounds, i.e., saturated/unsaturated fatty acids, triacylglycerols, cholesterol, cholesteryl esters and phospholipids.

We focused our attention to these sub-bands to assess the quantitative changes due to chemical structural modifications. We computed the area ratio using

(1) $$A_{R}=\frac{\sum A_{{CH}_{3}}}{\sum A_{{CH}_{2}}},$$

where *A_R_* represents the area ratio, $\sum A_{{CH}_{3}}$ is the sum of all sub-bands referred to as the CH_3_ stretching vibration modes, and $\sum A_{{CH}_{2}}$ is the sum of all sub-bands referred to as the CH_2_ stretching vibration modes.

The first step was to determine the *A_R_* values for the average spectra of the ETI-treated and non-treated patients, resulting in values of 3.17 and 3.42, respectively. After this, the *A_R_* was calculated for all the patients, and the values obtained were statistically analyzed. The sensitivity and specificity of the diagnostic test were determined to evaluate if Raman analysis could distinguish between the ETI-treated and non-treated patients. The ROC curve and Youden index were also determined.

**Figure 4:**
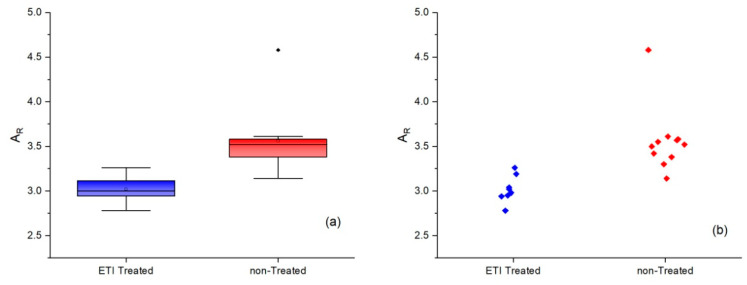
Box plot (**a**) and distribution graph (**b**) of the ETI-treated and non-treated patients.

**Figure 5:**
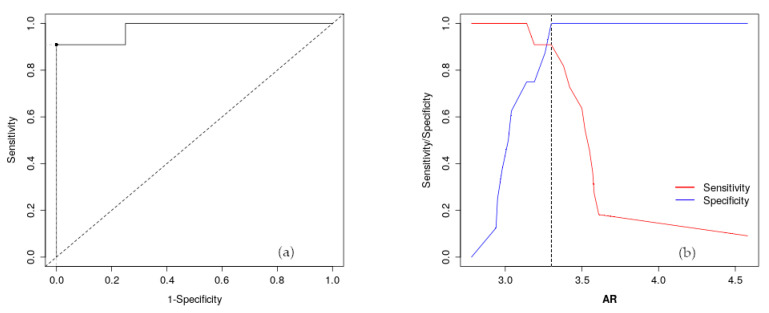
The ROC curve with the line of equality (dotted line) and the best cut-off point (black point) (**a**). The sensitivity and specificity vs. the *A_R_* value trend with the best cut-off point (vertical dotted points) (**b**).

The results of the statistical analysis are depicted in Figure 4 and Figure 5. Figure 4a accurately and reliably describes the discrete distribution of data by using a plot box graph. The box represents the first and third quartile of the data. Inside it, the median value is drawn as a horizontal line. The whiskers only extend to the most extreme observations within 1.5 of the difference between the third and first quartiles. The observed data points outside the boundary of the whiskers are plotted as outliers. For all the analyzed patients, the *A_R_* is reported in Figure 4b as a scatter plot.

Figure 5a shows the ROC curve calculated for the *A_R_* for all patients enrolled in this preliminary study. In the same figure, the line of equality and the optimal cut-off point are depicted. Figure 5b shows the trend of sensitivity (red line) and specificity (blue line) vs. the *A_R_* value. The optimal cut-off point is displayed as a vertical dotted line. The optimal cut-off point, calculated following the Youden method, was equal to 3.30.

The accuracy of the statistical analysis was evaluated calculating the Area Under Curve (AUC) value for the *A_R_*, and the AUC value is reported in Table 2. In the same table, the sensitivity and specificity are also indicated. The lower and upper limits for all the parameters, which represent the 95% confidence interval, are also reported.

## 3. Discussion

The present study was performed with the objective of determining the potential of Raman spectroscopy to differentiate the response of patients to ETI treatment, which represents the most recent therapy available for CF disease.

The starting point was the analysis of the serum samples from the ETI-treated and non-treated patients. All the obtained spectra exhibited the main typical protein vibrational modes. The disulfide band was centered at ~520 cm^−1^ [33], the band at ~760 cm^−1^ can be assigned to tryptophan [38], and the tyrosine doublet peaks were present at ~830 cm^−1^ and ~850 cm^−1^ [34,35]. The peak at about 1003 cm^−1^ can be assigned to phenylalanine [36]. The bands at ~1300 cm^−1^ and ~1450 cm^−1^ can be assigned to amide III vibration and CH_2_ scissoring deformation, respectively [37,38]. The amide II and amide I vibration bands were located at 1550 cm^−1^ and 1650 cm^−1^, respectively [39,40]. The large band centered at ~2935 cm^−1^ can be associated with C-H stretching vibration [41]. Our attention was focused on this band, as the main difference between the mean spectra of patients treated ETI and those not treated was qualitatively evident in this band. In particular, to assess the quantitative changes due to chemical structural modifications, we computed the area ratio as expressed in Equation (1). Based on the literature, the involved sub-bands could be associated with the CH_3_ and CH_2_ symmetric and anti-symmetric stretching of lipids. We observed a decrease in the *A_R_* value when the CF patients were treated with ETI. This behavior suggested a change in vibrational modes, which could be associated with lipid conformational modifications induced by a decrease in the inflammatory status, implying an active role of ETI in restoring the imbalance of lipids.

Statistical analysis showed an AUC value of 0.977, which can be considered outstanding. Sensitivity and specificity values were 0.909 and 1, respectively, with a 95% confidence level. The optimal cut-off point to maximize the Youden index was 3.30.

It is interesting to note that impaired CFTR function leads to perinuclear free cholesterol accumulation [52] and, at the same time, this perinuclear cholesterol accumulation is reversible. The ETI treatment seems to precisely act on this lipid imbalance to promote a repairing action. We would like to highlight that the response to treatment is not the same in all patients and the rate of adherence to treatment is variable. In addition, in clinical practice, a reduction in the effectiveness of ETI was taken into consideration. From all the considerations, we explored, through Raman spectroscopy, the possibility of finding a rapid, inexpensive test that did not require any additional effort from the patient and could identify their response to treatment.

The obtained results must be seen prospectively; the observed variation in the *A_R_* values can be associated with different clinical situations, such as differences in patient response rates to the drug and/or in evaluating adherence to treatment.

## 4. Materials and Methods

### 4.1. Patients and Serum Samples

Patients were selected during a routine visit to the Cystic Fibrosis Unit, University Hospital G Martino, Messina.

To perform this preliminary study, the observations did not imply any further evaluation for the patient other than routine clinical assessment. In fact, for our research, the analyzed serum samples were the same as the ones used during the clinical evaluation. Informed consent was obtained from all patients before performing the test.

We selected 8 patients on ETI treatment for at least one year. As controls, we selected 11 patients with genotypes unresponsive to ETI. In order to better evaluate the difference between the treated and non-treated patient, assuming that this difference is dependent on a reduction in systemic inflammation in the treated patients, we excluded patients with pulmonary exacerbations. It is well known that in CF pulmonary exacerbations are associated with higher levels of inflammation biomarkers, which could invalidate the results of our pilot study [53,54]. In Table 3, the demographic and clinical features of the patients are presented. As a measurement of treatment efficacy and therapeutic adherence, patients treated with ETI underwent sweat chloride determination, a robust endpoint of ETI efficacy. For non-treated patients, we considered the most recent sweat chloride determination.

For each patient, a whole-blood sample was collected into 5 mL vacuum tubes and then separated by centrifugation at 3000 rpm for 10 min at room temperature. A total of 2 mL of supernatant (serum) was collected. The obtained serum samples were stored in a refrigerator at 0–4 °C until use in Raman analysis.

### 4.2. Raman Spectroscopy Analysis

All serum samples were analyzed within 6 h after collection. Raman analysis was performed using a DXR-SmartRaman Spectrometer (Thermo Fisher Scientific, Waltham, MA, USA) equipped with a diode laser with an excitation wavelength of 785 nm. Before each measurement, the DXR-SmartRaman Spectrometer was calibrated using standard samples of known wavenumber provided by the manufacturer.

To acquire the Raman spectra, the 180-degree sampling accessory for the DXR-SmartRaman Spectrometer was used, and 100 μL of serum was pipetted into a vial; the vial was then placed into the sample holder. All the samples were irradiated with a 24 mW excitation laser (at the sample location) leaving a 50 µm spot pinhole, and the CCD collected the Raman signals with a grating of 400 lines/mm. The Raman measurements were conducted over a wavenumber range of 400–3300 cm^−1^ with a resolution of about 2.0 cm^−1^, as specified by the manufacturer.

To obtain high signal-to-noise ratio (S/R) spectra, each Raman spectrum was acquired for 30.0 s and averaged over 16 accumulations. The total acquisition time was 8 min for each spectrum. On average, three spectra were acquired from each serum sample.

For each spectrum a spline baseline correction was performed to reduce the attenuation of the weak Raman scattering signal, and the obtained spectrum was then normalized to the phenylalanine peak band, centered at about 1003 cm^−1^, which is typically intense and relatively isolated from any other peaks. Here, it showed no interference from neighboring peaks. Usually, the Phe ν-ring peak is used as an internal standard, as it has been reported to be insensitive to the micro-environment and exhibits the absence of interference with other peaks and bands [36].

Additionally, the normalization choice has an important impact on the vibrational features highlighted for their contribution in the spectral variability of a data set. In all cases, normalization was performed to cancel out the effect of laser fluctuation and the heterogeneity of the sample configuration on the mirror. Spectra and biochemical assays have demonstrated that biological sample peaks (not bands) are mostly stable, with phenylalanine being the most stable peak (1003 cm^−1^) [55]. Due to the stability of this peak, normalization of the spectra to the phenylalanine peak is common in Raman spectroscopy [56]. For this reason, we chose “peak normalization”, unlike other methods, because the Phe peak is insensitive to external reactions.

The obtained spectra were classified into two groups as follows: ETI-treated and non-treated. For each group, the average spectrum was determined. The difference spectrum of the CF patients was also computed by subtracting the average spectrum of the ETI-treated patients from the average spectrum of the non-treated patients.

The main difference between the mean Raman spectra of the ETI-treated and non-treated patients was observed in the 3020–2800 cm^−1^ wavenumber range. For this reason, our attention was focused on this region, and the experimental data were fitted using the functions available on the Omnic for dispersive Raman 9.0 software. Starting from the assumption that a serum Raman spectrum band can be considered as the linear sum of fundamental elements of the secondary structure, a quantitative analysis of the secondary structure was performed, and the second derivative was computed on the fitted curves of the spectra using the OriginPro 9.0 software (OriginLab Corporation, Northampton, MA, USA). The analysis of the second derivative was important, as it permitted a preliminary indication of the minimum number of band components and their peak positions, according to a procedure already successfully applied in the analysis of Raman serum spectra [57].

In fact, the computed minima in the Raman spectra derivative profiles represent the center frequencies of the sub-bands.

One of the main advantages of the analysis of the *n*-th derivative, and, in particular, the second derivative, is the possibility to perform this action without arbitrary deconvoluted parameters.

To distinguish between superimposed and very close bands and reducing band noise, the above band was deconvoluted using a Voigt profile with 10 cm^−1^ bandwidths.

The Voigt profile, consisting of a mixed convolution of Gaussian and Lorentzian curves, represents a good starting point for liquid sample analysis.

The CH_3_ to CH_2_ ratio was used to evaluate changes due to chemical structure modifications.

### 4.3. Statistical Analysis

To evaluate the validity of Raman spectroscopy for the ETI-treated and non-treated respondence, the sensitivity and specificity of the diagnostic test were determined by clinical evaluation, as this is considered the gold standard in the evaluation of pharmacological responses. The ROC curve and its related area under the curve were also determined. They represent the plot of sensitivity vs. 1-specificity and overall accuracy of the test, respectively. In this study, sensitivity referred to the ability of Raman spectroscopy to correctly identify ETI-treated patients; conversely, specificity referred to the ability of Raman spectroscopy to correctly identify non-treated patients.

The optimal cut-off point was determined using the Youden index [58], which represents the maximum vertical distance or difference between the ROC curve and the diagonal. This is used to choose the optimal threshold value or cut-off point for a biomarker of interest. In this study, we used it to measure the effectiveness of Raman spectroscopy in differentiating between ETI-treated and non-treated patients.

## 5. Conclusions

A growing number of clinical studies have demonstrated the efficacy of ETI therapy and its ability to significantly reduce pulmonary and gastrointestinal manifestations, sweat chloride concentration, exocrine pancreatic dysfunction, and infertility/subfertility, among other disease signs and symptoms [59,60,61]. Among the markers of lipid metabolism, cholesterol levels have been shown to notably improve following one year of ETI treatment. Likewise, the fat-soluble vitamins D and E, known to be deficient in CF patients, tend to return to their normal levels in individuals undergoing ETI therapy [62].

On the other hand, little is known about ETI non-responders, and when side effects occur or a poor adherence is suspected, it is difficult to monitor drug levels and define a safe drug threshold level [63]. Finding a marker for EI activity based on the anti-inflammatory response could be helpful in these scenarios.

Even though the number of enrolled patients was small, the preliminary results are very encouraging.

The obtained results demonstrate that Raman spectroscopy could allow us to evaluate the response of CF patients to ETI treatment and the rate of adherence, providing important information about the active role of ETI in restoring lipid imbalance.

We are fully aware that the low number of patients does not allow us to draw definitive conclusions, and thus our future efforts will be oriented towards expanding the number of patients. At present, the only truly effective test used for the response evaluation to treatment (other than a full clinical evaluation) is the measurement of sweat chloride levels. This test is not easy to carry out and requires a certain amount of time. In this context, Raman spectroscopy could represent an additional, rapid, and inexpensive test that could be combined with routine laboratory methods to obtain diagnostic information.

Other differences in the Raman spectra, which we were unable to exclude, could become more evident when a larger patient cohort is considered. For this reason, our intention is to continue increasing the number of patients and extend Raman spectroscopy to ETI-treated patients failing to response to treatment, as well as enrolling patients with pulmonary exacerbations.

## Figures and Tables

**Table 1:** Raman peak position, tentative assignment and related reference of the nine sub-bands obtained from the Voigt deconvolution of the 3020–2800 cm^−1^ band.

Raman Peak Sub-Band (cm^−1^)	Assignment	References
2858	CH_2_ symmetric stretch	[42,44]
2877	CH_3_ symmetric stretch	[45]
2897	CH_2_ anti-symmetric stretch	[45]
2912	C-H vibrational mode	[46]
2924	CH_2_ anti-symmetric stretch	[42,47]
2940	CH_3_ symmetric stretch	[42,48]
2960	CH_3_ anti-symmetric stretch	[42,49]
2977	CH_3_ symmetric stretch	[50]
2984	CH_2_ group vibration	[51]

**Table 2:** The AUC, sensitivity, specificity, and confidence intervals (CI) for the statistical analysis.

	Value	CI
AUC	0.977	0.924–1
Sensitivity	0.909	0.587–1
Specificity	1	0.631–1

**Table 3:** The demographic and clinical features of the tested patients.

	Treated	Non-Treated
Age (mean ± SD)	22.4 ± 6.3	26.2 ± 8.5
F/M	2:3	4:1
FEV1 ^1^ (mean ± SD)	104.4 ± 22.8	87.2 ± 18.9
BMI ^2^ (mean ± SD)	20.9 ± 3.7	22.4 ± 3.9
C-reactive protein ^3^ (mean ± SD)	0.50 ± 0.44	0.78 ± 0.52
Sweat chloride ^4^ (mean ± SD)	42.4 ± 13.4	86.6 ± 19.8

^1^ forced expiratory volume per 1 s. ^2^ body mass index expressed as kg/cm, ^3^ expressed as mg/L, and ^4^ expressed as mEq/mg.

## Data Availability

The data generated or analyzed during this study are available from the corresponding author on reasonable request.
